# Iron and Vitamin A Status of Children Aged 0 to 36 Months in Thulamela Municipality, Vhembe District, South Africa †

**DOI:** 10.3390/children11081018

**Published:** 2024-08-20

**Authors:** Anzani Mugware, Selekane Ananias Motadi, Alphonce Bere, Lindelani Fhumudzani Mushaphi

**Affiliations:** 1 Department of Nutrition, Faculty of Health Sciences, University of Venda, Private Bag x5050, Thohoyandou 0950, South Africa; selekane.motadi@univen.ac.za (S.A.M.); lindelani.mushaphi@univen.ac.za (L.F.M.); 2 Department of Mathematical and Computational Science, Faculty of Science, Engineering and Agriculture University of Venda, Thohoyandou 0950, South Africa; alphonce.bere@univen.ac.za

**Keywords:** iron, vitamin A, anaemia, children, anthropometric status

## Abstract

**Objective:** The present study assessed the iron and Vitamin A status of children aged 0 to 36 months in Thulamela municipality, Vhembe District. **Methods:** A cross-sectional study was conducted among 250 children aged 0 to 36 months attending well-baby clinic services with their mothers. Convenience sampling was used to select study participants, and simple random sampling was used to choose clinics. Data were gathered via a questionnaire administered by the researcher and field workers from August to September 2019. Standard techniques were used to measure body weight and height. In addition, serum retinol, haemoglobin, iron, ferritin, transferrin saturation, and transferrin levels were also assessed. Information on dietary diversity was gathered through a 24 h dietary recall. **Results:** The prevalence of underweight, wasting, and stunting was 3.6%, 2%, and 9.2%, respectively. Using serum retinol <10 µg/dL, 22% of children had vitamin A deficiency. The prevalence of anaemia was 53.6%, while 13.1% of children had iron deficiency anaemia when using serum ferritin of less than 12 μg/dL. Most children (90.8%) had an inadequate dietary diversity score, while 9.2% had sufficient dietary diversity. The most consumed food groups were grains, roots and tubers, vitamin A rich fruits and vegetable, and flesh foods. A higher percentage (44%) of children with low iron ferritin were underweight compared to those with normal iron ferritin (df = 1, *p*-value = 0.007). **Conclusion:** Iron, anaemia, and vitamin A deficiencies, accompanied by a high prevalence of stunting, were common among children in Thulamela Municipality. Thus, improving nutritional status in this area is a critical need.

## 1. Introduction

The first two years of life are a vital period to promote the growth and development of infants and young children [1]. The period from birth to two years is considered as the critical period in which malnutrition can occur [2,3]. Promotion of optimal feeding practices and meeting micronutrient requirements are crucial steps in combating malnutrition, particularly at this age [4]. According to the World Health Organization, [5] malnutrition includes overnutrition, which is an excess of nutrients, and undernutrition, which refers to a deficiency in essential nutrients. Malnutrition has a negative impact on the health status, growth, and development of infant and young children, especially in the first two years of life [6]. Malnutrition may result in long-lasting and irreversible consequences, such as impairment of growth and development and poor resistance to infections [7,8].

Despite the introduction of food fortification in 2002 and vitamin A supplementation strategies, micronutrients remain persistent in South Africa [9]. These micronutrient deficiencies may be coupled with protein or energy malnutrition and occur as part of a cycle of malnutrition. Children under five years of age are the most vulnerable group for these micronutrient deficiencies [10,11]. The deficiency of micronutrients is more common in countries where the dietary diversity required to achieve micronutrient requirements is inadequate and the diets are energy-dense and monotonous. Children need iron for appropriate behavioural organisation as well as for the development of their cognitive and psychomotor development [12]. According to Bailey et al. [13], vitamin A is crucial for physiological processes in the body, including survival, bone formation, eyesight, cognition, and the integrity of epithelial cells. Vitamin A is important for reducing the risk of common infections such as diarrhoea and measles [13].

South Africa, like other developing regions, faces the burden of malnutrition in the form of undernutrition [14], a public health concern affecting vulnerable population groups, especially children under the age of five years [15]. The increasing rates of micronutrient deficiency in children in South Africa are attributed to various factors, such as food insecurity, poor feeding practices, childhood illnesses, and poor access to water and sanitation [16]. A substantial risk of malnutrition exists in the early stages of life, particularly in the first 1000 days of life, when improper feeding practices for infants and young children are combined with recurrent illnesses and infections [8,17]. According to Sokhela et al. [18], malnutrition and micronutrient deficiencies are prevalent in South Africa due to the early introduction of solids before six months of age and the inadequate consumption of protein-rich diets.

In South Africa, 1 capsule of 100,000 IU is recommended for children aged six months, 1 capsule of 200,000 IU for those aged twelve months, and 200,000 IU for those aged eight months. From 24 months, every child should receive 1 capsule of 200,000 IU of vitamin A. Furthermore, oral mebendazole (100 mg) is administered to children 1–2 years of age, 12 h a day for 3 days, and to children above 2–5 years of age, 500 mg in one dosage [19].

The results of the South African National Health and Nutrition Examination study 1 and the Demographic and Health Survey study among children showed that 21.4% and 61.3%, respectively, were anaemic [20,21]. Despite the fact that numerous nutritional studies involving South African children have been carried out [22,23,24], little is known about the prevalence of iron and vitamin A deficiencies among children living in rural areas. In view of this, we carried out this study to assess the iron and vitamin A status of children aged 0–36 months living in a rural region of Thulamela Municipality in Vhembe district. According to a study by Tshivhase et al. [25], there are a number of obstacles that prevent children in this district’s clinic from obtaining child health services, which makes it more difficult to meet Sustainable Development Goal (SDG) 3.

Thulamela Municipality has approximately 2234 children who are attending well-baby clinic services [26]. The municipality has well-dispersed PHC facilities. Health care services in Thulamela Municipality are delivered by 49 clinics, 3 community health centres, 2 district hospitals, and 15 mobile services [27]. This municipality was chosen for this study because it is considered the Eden of the Limpopo province due to the vast yield of fruits and vegetables [28]. The municipality’s subsistence farming is mostly reliant on rainfall. During the ploughing season, rural communities gather enough bags of maize meal to last them for many years. After harvest, these bags of corn are transported to the adjacent milling stations where they are processed into maize meal without any fortification. Due to this, the population’s access to fortified foods which could provide the micronutrients needed for children’s growth and development is restricted.

## 2. Methods and Materials

### 2.1. Study Design and Setting

The study was conducted in Thulamela Municipality, which is one of the municipalities of Vhembe District. The district is divided into four municipality, namely Thulamela, Collins Chabane, Makhado, and Musina. Simple random sampling was used to select the clinics. A list of clinics was obtained from the Department of Health, Vhembe District, and each clinic was assigned a number, 18 of which were randomly selected for inclusion in the study. Convenience sampling was used to select municipality and study participants. Children who were aged 0 to 36 months and whose parents consented and were present on the day of data collection were included in the study. Data were collected by the researcher and field workers (Nutritionist and Phlebotomist) from August to September 2019. In this district, fruits and vegetables are available throughout the years. However, a number of them are available during the spring and summer.

### 2.2. Sample Size and Sample Technique

The sample size was calculated using Solvin’s formula (n = N/(1 + Ne^2^), using a population size of 2234 children attending well-baby clinic services. A tolerance for error of 0.05 and a 95% confidence level were used. The formula yielded 340 subjects; an addition of 10% was added for attrition. A total of 250 children were selected to participate in the study after obtaining written consent from their parent/caregivers. The same formula was used to calculate the number of clinics to be visited from 43, and the formula yielded 39 clinics. Data were collected from 18 clinics in Thulamela Municipality. The total number of clinics and participants were reduced due to financial constraints. To determine the number of clinics used per cluster, the number of clinics was divided by the number of clusters (18 clinics/6 clusters = 3 clinics per cluster). To determine the number of participants used per clinic, the number of participants was divided by the number of clinics (250 participants/18 clinics = 14 participants per clinic). During data collection, some of the clinics had less than the required sample size, while others had more than the required sample size. Blood samples were only collected from 138 children because some of the mothers refused to give consent for drawing blood.

### 2.3. Variables Measured

Variables measured were socio-demographic characteristics, anthropometrics (weight, length, height, and mid–upper-arm circumference), biochemical measurements of iron status (serum iron, ferritin, transferrin, and transferrin saturation) and vitamin A status (serum retinol concentration), and dietary diversity. Using questionnaires administered during an oral interview with parents or caregivers, the socio-demographic characteristics were assessed.

### 2.4. Anthropometrics

Anthropometric assessments were performed according to standard procedures [29]. The measurements were taken in duplicate using calibrated equipment with the children wearing light clothing and no shoes. Height was measured to the nearest 0.1 cm using a calibrated portable stadiometer. The length was measured to the nearest 0.1 cm using a measuring length board, and weight was measured to the nearest 0.01 kg on a portable Seca solar scale (model 0213) (Seca, Hammer Steindamm, Hamburg, Germany). Prior to taking measurements, the solar scale was calibrated using a known weight.

The World Health Organization’s (WHO) child development standards were used to interpret the anthropometric status [30,31]. According to the WHO [31], the Z-score classification cutoff points were ≤−2 SD for Weight for Age, Weight for Height, and Height for Age. These are referred to as underweight, wasting, and stunting, respectively. Severe stunting, severe wasting, and severe underweight are classified according to the cutoff criterion of ≤−3 SD. Moreover, the cutoff points for weight for height are as follows: <+3 SD is considered overweight, while >3 SD is considered obesity.

### 2.5. Biochemical Analysis

A phlebotomist from the Ampath Pathology Laboratory collected 138 blood samples from 18 clinics in Thulamela Municipality. The mother helped the professional paediatric nurse immobilise the youngster. As a result, the tourniquet was applied to the child by the professional paediatric nurse approximately two finger widths above the site of the venepuncture. Well-fitting, non-sterile gloves were worn by the professional paediatric nurse. The collection area was cleaned, then left to dry. A skilled paediatric nurse pulled the skin taut with her thumb two finger widths below the location of the venepunture, then fully pressed the vacuum tube onto the needle. Blood started to pour into the tube, filling it up until the hoover was lost or the tube was completely full. After 5 millilitres of blood was taken, the tourniquet was loosened. After covering the venepunture site with a dry gauze, the needle was carefully removed. Moms were urged to submit their applications again. The samples were transported on dry ice to the laboratory for subsequent analysis. The blood samples were analysed using standard procedures in the Ampath Pathology Laboratory (Drs Du Buisson, Kramer Inc./Ing, Pretoria, South Africa). The Ampath Pathology Laboratory is SANAS (South African National Accreditation System, Pretoria, South Africa)-accredited. The blood samples were used to assess the vitamin A and iron status of children.

### 2.6. Definitions of Iron and Vitamin A Deficiencies and Anaemia

Anaemia was defined as Hb levels < 11 g/dL, while Hb level 10.0–10.9 g/dL was classified as mild anaemia, a Hb level of 7.0–9.9 g/dL was defined as moderate anaemia, and Hb levels < 7.0 g/dL were classified as severe anaemia for the children [32]. Iron deficiency was defined as serum ferritin (SF) < 12 mg/L or TSAT < 15% [33]. Hb concentrations < 7 g/dL were considered as severe anaemia, 7 to 9.9 g/dL as moderate anaemia, and Hb >10–<11 g/dL as mild anaemia [33], whereas vitamin A deficiency was defined as serum retinol concentration < 10 μg/dL [34]. Iron-deficiency anaemia (IDA) was defined as having low Hb levels accompanied by low TSAT or SF or both [32,33]. Serum transferrin levels below 1.0 g/L were classified as severe depletion, and 1.5–2.0 g/L as depletion. Serum iron below 40 μg/dL was classified as iron depletion, and <60 μg/dL was classified as mild depletion.

### 2.7. Dietary Assessment

The 24 h dietary recall method was used to assess the dietary diversity of children who were already introduced to complementary foods. On the day of the growth-monitoring visit, a 24 h dietary recall was carried out in accordance with their Road to Health Booklet. Clinics varied on the date, but usually on Tuesday and Wednesday.

Mothers were asked to recall all the food that the child consumed during the previous 24 h. The researcher used food cards to assist mothers to remember the food items they fed their children in the previous 24 h. The Minimum Dietary Diversity (MDD) indicator was based on the following seven food groups: (a) grains, roots, and tubers; (b) legumes and nuts; (c) dairy products (milk, yoghurt, and cheese); (d) flesh foods (meat, fish, poultry, and liver/organ meats); (e) eggs; (f) vitamin A-rich fruits and vegetables; and (g) other fruits and vegetables [35]. However, the dietary diversity of those who were exclusively breastfeeding was not assessed, hence the use of seven food groups. The minimum dietary diversity (MDD) score was interpreted using the WHO-recommended cut-off point, with a value of “1” indicating if the child had consumed four or more groups of foods and “0” if less [36]. The score ranged from 0 to 7. The dietary diversity score was deemed poor if it was less than 4, and as adequate if it was greater than 4.

### 2.8. Ethical Considerations

The University of Venda Research Ethics Committee granted ethical permission (SHS/19/NUT/01/1503, 23 May 2019). The Provincial Department of Health Research Committee gave its approval to the project. The study was performed in accordance with the principles of the Declaration of Helsinki [37], Good Clinical Practices, and the laws of South Africa. The mothers and guardians received an oral and written explanation of the study, including any potential risks and benefits. A signed informed consent form was given to mothers and legal guardians so they could sign for both themselves and the child.

### 2.9. Statistical Analysis

Data were analysed using Statistical Package for Social Sciences (SPSS, Chicago, IL, USA) version 29. Descriptive statistics were computed on the data, and the mean and standard deviations were used to describe continuous data as the data were normally distributed, while frequencies were used to describe categorical data. The Spearman correlation and chi-square tests were used to investigate the associations between micronutrients and anthropometric indicators. A *p* < 0.05 was considered to declare a result statistically significant.

## 3. Results

### 3.1. Socio-Demographics

The Mean (±SD) age of the study participants in months was 10.53 (±8.39). Of all the children, 50.8% were between the age of 6 and 24 months. Most (52.2%) of the participants were boys, while 47.8% were girls. Less than half (49.2%) of the mothers were between the age of 20 and 29 years old. The majority (54%) of the mothers were single, while 37.6% were married. The majority (96.4%) of the mothers had a high literacy level, while the minority had a low literacy level. Three-quarters of the mothers were unemployed (Table 1).

### 3.2. Anthropometrics Status of Children

The prevalence of underweight, wasting, and stunting was 3.6%, 2%, and 9.2%, respectively. Of all the children, 16.4% were mildly underweight, while 19.2% were mildly wasted, and 23.6% were mildly stunted. About 6.5% were at risk of malnutrition according to their mid–upper-arm circumference (MUAC) cutoffs, and 1.5% had moderate acute malnutrition. When using BMI for age, 16% of children were at possible risk of being overweight, while 1.6% were overweight (Table 2).

### 3.3. Iron and Vitamin A Status of Children

**Figure 1:**
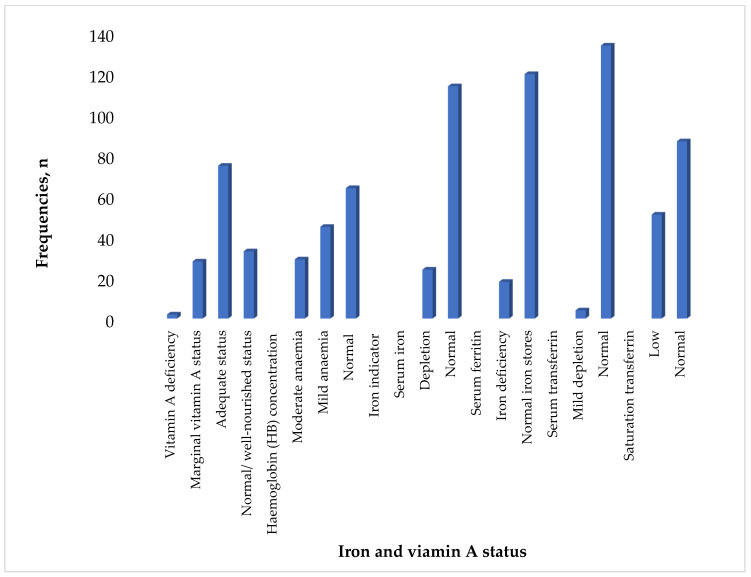
Iron and vitamin A status of children (n = 138).

The findings of this study suggest that 22% of the children had vitamin A deficiency when using serum retinol <10 µg/dL. The prevalence of anaemia was 53.6%, while 13.1% of the children had iron deficiency anaemia when using serum ferritin of less than 12 μg/dL. Using a haemoglobin concentration of <7.0–9.9 g/dL as an indicator of anaemia, 21% of the children had moderate anaemia, and 32.6% had mild anaemia (10.0–10.9 g/dL). Serum iron levels less than 60 g/dL indicated that 17.4% of the children had low iron levels. When using serum ferritin less than 12 μg/dL, 13.1% of all the children were found to be iron-deficient. Additionally, 2.9% of the children exhibited mild depletion using serum ferritin levels between 1.5 and 2.0 g/L. Only 20.3% of the children had marginal vitamin A status when using serum retinol concertation of 10–19.9 μg/dL, while 1.4% had vitamin A deficiency when using serum retinol concentration of <10 μg/dL (Figure 1).

### 3.4. Dietary Diversity of Children

**Figure 2:**
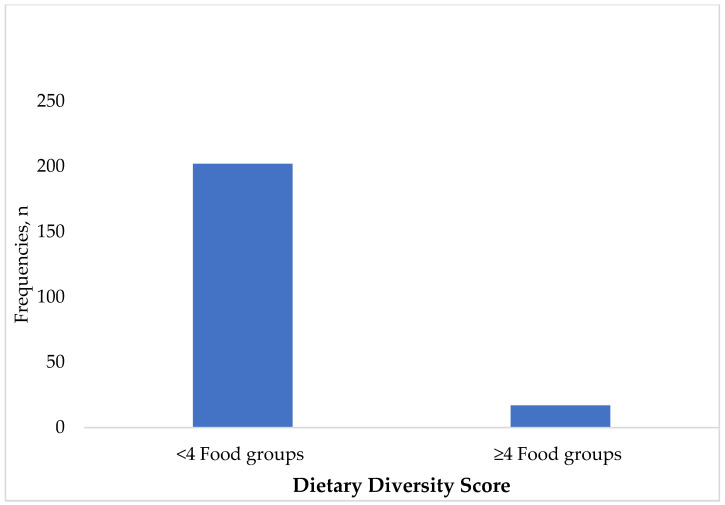
Dietary diversity of the children (n = 219). Breastmilk was also excluded from the calculation of dietary diversity score.

Most children (90.8%) had inadequate dietary diversity scores, while 9.2% had sufficient dietary diversity. Majority of the children (219, 87.5%) were introduced to complementary feeding before the age of six months, while 7.6% (19) of the children were exclusively breastfed, and only 4.9% (12) were formula-fed (Figure 2).

**Figure 3:**
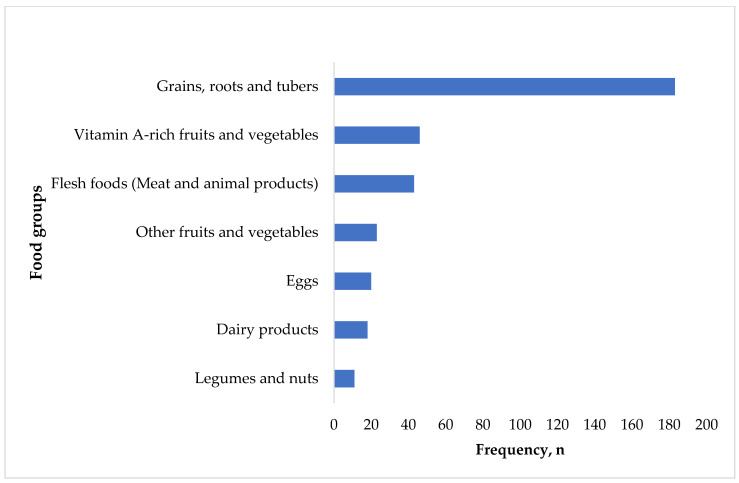
Frequency of food group consumption.

The most-consumed food groups were grains, roots, and tubers, The least-consumed food groups were vitamin A-rich fruits and vegetables, flesh foods, legumes and nuts, dairy products, eggs, and other fruits and vegetables (Figure 3).

### 3.5. Association between Anthrpometric and Biochmical Measurements

Of all the children whose blood was collected, weight-for-length (WLZ) was negatively correlated with transferrin (r = −0.126; *p* = 0.032) but positively correlated with transferrin saturation (TSAT) (r = 0.154; *p* = 0.053) and ferritin (r = 0.123; *p* = 0.000). Weight-for-age was positively correlated with Hb (r = 0.169; *p* = 0.047) and ferritin (r = 0.148; *p* = 0.054) but negatively correlated with transferrin (r = −0.016; *p* = 0.853). Length-for-age was positively correlated with transferrin (r = 0.158; *p* = 0.054). BMI-for-age was negatively correlated with transferrin (r = −0.213; *p* = 0.016) but positively correlated with TSAT (r = 0.121; *p* = 0.024) and ferritin (r = 0.216; *p* = 0.036) (Table 3).

### 3.6. The Association between Micronutrients and the Anthropometric Status of Children

A higher percentage (44%) of children who had low iron ferritin levels were underweight, as compared to those who had normal iron ferritin levels (df = 1, *p*-value = 0.007). Only 18% of the children with normal ferritin levels were underweight. There was a significant association between iron ferritin and length-for-age (df = 1, *p*-value = 0.030). About 22% of children with low iron ferritin levels were stunted (Table 4).

## 4. Discussion

In the South African Thulamela Municipality of the Vhembe District, anaemia, iron and vitamin A deficiencies, coupled with low dietary diversity, are problems. This occurred despite the implementation of many initiatives, such as food fortification and vitamin A supplementation programmes, aimed at addressing undernutrition and micronutrient deficiencies. The prevalence of these micronutrients is higher than those reported in the national surveys by Shisana et al. [20] and NDoH, Stats SA, SAMRC, and ICF [21]. The results warrant immediate action since the prevalences of micronutrients was higher than 20%, which is regarded as a public health concern [32,33]. This might be because children were given soft maize-meal porridge served with water, without being given any additional nourishment. The levels of micronutrient deficiency in this investigation suggest that children were not receiving the basic minimum nutrition needed for optimal growth and development. One cause of micronutrient deficiency is long-term nutritional deprivation, and its consequences are associated with delayed mental development [7,38], poor school performance [8,39], and reduced intellectual capacity [40].

The current research exhibited a low number of children being exclusively breastfed with a high number being introduced to complementary feeding. In 2008, the WHO used seven food groups to measure children’s dietary quality and did not capture breast milk as a food source. In evaluating the quality of their diets, this has chastised breastfeeding children, relative to formula-fed children. The 2021 guidelines include breast milk as one of the eight food groups, which improves the accuracy of the MDD comparison between infants who are breastfed and those who are not [41]. However, the current study used the old seven food groups because most of them were already introduced to complementary foods. The exclusion of breastmilk in assessing the dietary diversity in the current study was due to the fact that the current study was conducted in 2019, prior to the introduction of the WHO guideline [41] which included breast milk into dietary diversity, increasing the score to 5 out of 8. It is noteworthy that the use of the seven food groups to assess dietary diversity in the current study instead of eight could be the reason why most children had poor dietary diversity.

Using the seven food groups, the children in this study had poor dietary diversity. This could be attributed to the fact that the study was collected in a rural village of a municipality where their diet is predominantly made up of starchy foods and a minimal amount of fruits and vegetables, with no animal food. Most low- and middle-income children suffer from nutrition-related public health problems as a result of their repetitive diets being heavy in starches and cereals [42]. The findings of this study are consistent with reports in Vhembe District [22,23,24] and other part of South Africa [42,43]. The poor dietary diversity is the reflection of the quality of their diet and how monotonous their diet was. The poor dietary diversity could be due to fact that majority of mothers were unemployed, and most households were dependent on child grants, which are not enough to purchase the food needed for growth and development.

The results of the current investigation showed a lower prevalence of underweight, wasting, and stunting among children [24]. The poor nutritional status of the children is attributed to the poor dietary diversity score, which has been shown by the study participants in the current study. Monotonous diet and poor feeding practices are directly linked to undernutrition during childhood [44,45,46]. The current nutritional status among children was lower than those documented in SANHANES-1 by Shiana et al. [20] and in NDoH, Stats SA, SAMRC, and ICF [21]. The adverse effects of poor nutritional status include recurring illness, weakness, delayed physical and mental development, irritability, poor appetite, and low-weight-for-age, while the long-term adverse effects are short-height-for-age, poor learning ability, poor performance at school, and poor general health [47].

The results showed that WLZ correlated positively with TSAT and ferritin. Iron deficiency (ID) causes myoblast proliferation to be impaired, aerobic glycolytic capacity to be reduced, and signs of myocyte atrophy and apoptosis to be induced, all of which contribute to the loss of muscle mass [48]. WAZ positively correlated with Hb and ferritin. The results of this investigation are consistent with a study conducted in Nepal by Agho et al. [49], which found that anaemia and sTfR biomarkers were associated with underweight. LAZ correlated positively with transferrin. Several research have corroborated that iron deficiency, which leads to anaemia, is a contributing factor to poor growth [49,50]. Iron supplementation has also been shown to improve linear growth in children who are anaemic [49,50]. The requirement of iron increases during periods of rapid growth in infancy [51]. Inadequate intake of iron from their rich sources may lead to growth faltering, delayed development, and stunting in children from developing countries [52]. BMI/A positively correlated with TSAT and ferritin. Several investigations have found that individuals who are overweight or obese experience persistent subclinical inflammation, which may subsequently result in iron deficiency [53,54,55,56].

### 4.1. Implication of the Study

This study shows that although national programmes are put into place, they do not ultimately reach the most disadvantaged people in rural areas, particularly when maize is harvested and transported to nearby milling stations without any form of fortification. The milling process without fortification takes place despite the mandatory legislated fortification of flour and maize since 2003 [56]. These make most families lose out on additional micronutrients that could have helped them achieve their daily needs for these nutrients as a result of the process. The poor coverage of vitamin A supplementation of 44% in 2022 and poor consumption of vitamin A-rich foods played a significant role in the current prevalence of vitamin A deficiency in the current study. This means that parents are not bringing children for child-health services. The government should scale up vitamin A supplementation campaigns to increase coverage and promote growth-monitoring through early childhood development centres and home visits. The government should introduce mandatory iron supplements to children under the age of five in order to remedy the malnutrition.

### 4.2. Strength and Limitations

A limitation of the present study is that deworming was not measured, as it could have given us a better understanding of the prevalence of anaemia and iron deficiency. Furthermore, as this study was limited to the Thulamela Municipality in the Venda region, it is possible that its findings do not accurately represent behaviours across the provinces of South Africa and Limpopo. Due to financial limitations, inflammatory markers, including CRP and AGP, were not measured. The infection status of the children (e.g., HIV/AIDS) was not measured, and this could have affected the iron-level status. The mothers’ estimates were used to determine the diversity of diets. Although there may be a recall bias in these data, they may also accurately reflect daily consumption of the children. The current study used the WHO (2008) indicators for assessing infant- and young-child-feeding practices, which recommend the use of seven food groups to assess the dietary diversity score of children. These could have resulted in the underreporting of the dietary diversity of the children. Despite these limitations, the study reports on vitamin A status, which was unexpectedly higher given South Africa’s recently adopted vitamin A supplementation plan. The prevalence of Vitamin A was not expected as Vhembe District is considered the Eden of South Africa due to its vast yields of fruits and vegetables. The results of this investigation may serve as an indirect indicator of the coverage of vitamin A supplementation in this municipality.

## 5. Conclusions

The prevalence of anaemia, iron, and vitamin A deficiency among children in the rural areas of this municipality is a significant concern. In addition, the study found poor dietary diversity among children with vitamin A-rich fruits and vegetables, flesh foods, legumes and nuts, dairy products, eggs, and other fruits and vegetables being the least-consumed. The present study did not use the 2021 WHO guidelines, which recommend the inclusion of breast milk into the dietary diversity score. An association between anthropometric indicators and biochemical makers was also exhibited in this study. Nutrition professionals should intensify vitamin A campaigns not only at the clinic but also at the preschool level. The information on infant-feeding during child health services should emphasise the inclusion of fruits and vegetables after six months, as these are rich sources of vitamin A.

## Figures and Tables

**Table 1:** Socio-demographic characteristics of the study participants and their mothers (n = 250).

Variables	*n*	%
Child		
Age of child (months)	101	40.4
<6	127	50.8
6–24	22	8.8
>24		
Sex		
Boys	131	52.4
Girls	119	47.8
Mother		
Maternal age (years)		
≤19	31	12.4
20–29	123	49.2
30–39	88	35.2
≥40	8	3.2
Education status		
None	1	0.4
Low literacy level ^a^	8	3.2
High literacy level ^b^	241	96.4
Marital status		
Married	94	37.6
Single	153	62.4
Employment status		
Unemployed	188	75.2
Employed	62	24.8

^a^ primary school, ^b^ secondary and tertiary level.

**Table 2:** Anthropometric status of the children (n = 250).

Z-Score Classification	Interpretation	n	%	Mean ± SD
Weight for age				−0.24 ± 0.871
−3SD–≤2SD	Underweight	9	3.6	
−2SD–≤1SD	Mild underweight	41	16.4	
−1SD–+1SD	Normal	183	73.2	
>+1SD–≤+2SD	Possible growth problems	17	6.8	
Length-for-age				−0.35 ± 1.258
≤3SD	Severely stunted	1	0.4	
−3SD–≤2SD	Stunted	23	9.2	
−2SD–≤1SD	Mild stunted	59	23.6	
−1SD–<+3SD	Normal length	167	66.8	
Weight for length				−0.06 ± 1.049
−3SD–≤2SD	Wasted	5	2	
−2SD–≤1SD	Mild wasted	48	19.2	
−1SD–+1SD	Normal WHZ	161	64.4	
>+1SD–≤+2SD	Possible risk of overweight	33	13.2	
+2SD–<+3SD	Overweight	3	1.2	
BMI for age				−0.05 ± 1.064
−3SD–≤2SD	Wasted	6	2.4	
−2SD–≤1SD	Normal BMI/A	46	18.4	
−1SD–+1SD	Normal BMI/A	154	61.6	
>+1SD–<+2SD	Possible risk of overweight	40	16	
+2SD–<+3SD	Overweight	4	1.6	
MUAC for age				
11.0–12.5 cm	Moderate Acute Malnutrition	3	1.5	
12.5–13.5 cm	At risk of Acute Malnutrition	13	6.5	
>13.5 cm	Well-Nourished	184	92	

Descriptive statistics were used to analyse the data. BMI; body mass index, SD; standard deviation.

**Table 3:** Correlation between biochemical markers and anthropometric measurements.

Variables	HB	Iron	Transferrin	T Saturation	Ferritin	Serum Retinol
**WLZ**						
r	0.098	**0.007**	**−0.126**	**0.154**	0.123	0.034
*p*	0.255	**0.937**	**0.032**	**0.053**	0.000	0.690
**WAZ**						
r	**0.169**	0.109	−0.016	0.042	**0.148**	0.065
*p*	**0.047**	0.203	0.853	0.623	**0.054**	0.451
**LAZ**						
r	0.143	0.058	**0.158**	0.002	0.017	0.150
*p*	0.094	0.497	**0.054**	0.977	0.843	0.079
**BMI/A**						
r	0.091	0.032	**−0.213**	**0.121**	**0.216**	0.066
*p*	0.290	0.706	**0.016**	**0.024**	**0.036**	0.444
**MUAC-for-Age**						
r	0.101	0.081	−0.068	0.031	0.150	0.090
*p*	0.269	0.375	0.459	0.735	0.099	0.322
**HB**						
**r**	-	**0.176**	0.047	0.183	0.122	0.148
***p***	-	**0.039**	0.589	0.031	0.145	0.082
**Iron**						
**r**	**0.176**	-	0.025	**0.420**	0.020	0.112
***p***	**0.039**	-	0.775	**0.000**	0.814	0.193

Spearman correlation was used to test the association between biochemical markers and anthropometric measurements. BMI/A, body-mass-index-for-age; LAZ, length-for-age; TSAT, transferrin saturation; WAZ, weight-for-age; WLZ, weight-for-length. The bold values represent situations where there is a significant association.

**Table 4:** The association between micronutrients and anthropometric indicators.

Biochemical Indicators	Anthropometrics Indicators			
	Weight-for-Age		Df	*p*-Value
Iron Ferritin	Underweight	Normal	1	
Low	8 (44.4%)	10 (56.5%)		0.007 *
Normal	42 (18.1%)	190 (81.9%)		
	Length-for-age			
Iron Ferritin	Stunted	Normal	1	
Low	4 (22.2%)	14 (77.8%)		0.030 *
Normal	79 (34.1%)	153 (65.9%)		

* Chi-square tests were used to investigate the associations between micronutrients and anthropometric indicators.

## Data Availability

The datasets generated and analysed during the current study are not publicly available online because the university has the copyright. The datasets used during the current study are available from the corresponding authors on reasonable request.
